# Leveraging Social Media and Crowdsourcing to Recruit and Retain Military Veterans With Posttraumatic Stress Disorder or Experience of Harmful Gambling for mHealth Interventions: Descriptive Study

**DOI:** 10.2196/73706

**Published:** 2025-11-11

**Authors:** Conor Heath, Jess M Williams, Daniel Leightley, Dominic Murphy, Simon Dymond

**Affiliations:** 1 Centre for Military Gambling Research, Swansea University, Singleton Park, Swansea, SA2 8PP, United Kingdom, 44 1792205678; 2 Department of Population Health, Faculty of Life Science and Medicine, King's College London, London, United Kingdom; 3 King’s Centre for Military Health Research, Institute of Psychiatry, Psychology & Neuroscience, King's College London, London, United Kingdom; 4 Combat Stress Centre for Applied Military Health Research, Leatherhead, United Kingdom; 5 Department of Psychology, Reykjavík University, Reykjavík, Iceland

**Keywords:** military veteran, gambling, posttraumatic stress disorder, PTSD, acceptance and commitment therapy, ACT, mobile health, smartphone, social media, crowdsourcing, recruitment

## Abstract

**Background:**

Military veterans may be at increased risk of posttraumatic stress disorder (PTSD) compared to the general population. PTSD is often comorbid with harmful and problematic patterns of gambling. Behavioral therapies such as acceptance and commitment therapy have shown promise in treating these co-occurring disorders, especially if combined with mobile health (mHealth) interventions to circumvent known help-seeking barriers faced by veterans. However, to date, recruitment for mHealth interventions has been challenging and may impact intervention feasibility.

**Objective:**

In this paper, our objectives were to describe the strategies used to recruit UK military veterans with PTSD or experience of harmful gambling to a pilot study of a smartphone-based digital intervention, ACT Vet.

**Methods:**

We used several recruitment strategies, such as direct mailing, paid study advertising on social media (Facebook) and an online research platform (Prolific), study-specific website management, in-person event hosting with veterans’ charities, snowball sampling, and incentives for completion.

**Results:**

Results showed that, over 27 days, recruitment through Facebook accounted for 21 eligible veterans (n=7, 33% through unpaid advertising and n=14, 67% through paid advertising), whereas Prolific accounted for 50 veterans. Additional strategies recruited 8 eligible veterans. In total, 79 eligible military veterans were recruited for ACT Vet, with 24 (30%) completing the final steps of the study.

**Conclusions:**

Difficulties such as low advertisement conversion rate and participant and data attrition arose throughout this study. Our findings illustrate the relative effectiveness of social media– and online platform–based initiatives in recruiting veterans with PTSD or harmful gambling. Future research should consider establishing an online presence for effective digital intervention recruitment with diverse branding to attract representative samples of veterans for mHealth research.

## Introduction

Military veterans may face barriers when accessing help and support for difficulties related to mental health up to 11 years after service [1-3]. Among veterans, posttraumatic stress disorder (PTSD) is often prevalent alongside challenges related to anxiety, alcohol misuse, and depression that contribute to poorer well-being [4,5]. Moreover, veterans are also more likely to engage in and experience avoidance behaviors, including harmful gambling behaviors motivated by escape or avoidance of distress [6-9]. Recently, Dighton et al [9] highlighted that veterans may engage in gambling as a coping mechanism for distressing PTSD symptoms. They argue that increased gambling behaviors may lead to harmful gambling (gambling disorder), defined as problematic gambling behaviors that cause ongoing distress [10], and are likely to have an impact on the individual and the wider health and well-being systems that aim to provide support [11]. Therefore, there is an urgent need for improved access to efficacious treatment or interventions that may help manage comorbid mental health challenges such as PTSD and harmful gambling.

Promising therapeutic interventions for military veterans experiencing PTSD and comorbid harmful gambling include third-wave, mindfulness approaches such as acceptance and commitment therapy (ACT) [12-15]. ACT involves 6 core principles, defined as being present in the moment, identifying important values, committing to action, the self as context, cognitive defusion, and acceptance [16]. These principles focus on building psychological flexibility, the capacity to adapt and manage distress in accordance with valued goals [16,17]. There is also growing evidence of the feasibility and acceptability of mobile health (mHealth) interventions for PTSD, depression, alcohol misuse, and mental health [18-20]. Such apps embed forms of therapeutic support (eg, ACT) using smartphones, which allows for quick access to independent support supplemented by regular notifications, which may help with therapy engagement [21]. A meta-analysis by Linardon [22] revealed that smartphone-based apps could be effective in building acceptance and mindfulness skills (key elements of ACT). In addition, Farzandipour et al [18] suggested that apps may reduce self-reported PTSD symptoms. However, while Hawker et al [23] suggested that gambling-related apps could be accepted as an intervention, there is a lack of evidence-based treatment testing for many apps that are currently available [24,25]. Thus, evidence-based ACT mHealth apps may provide timely and easy-to-access help for military veterans with comorbid gambling-related harms and mental health difficulties.

When designing and evaluating interventions, feasibility trials, including those involving treatment for gambling in military veterans and nonveterans using mental health mHealth apps, tend to experience recruitment challenges and show high rates of attrition [26-28]. Combined recruitment strategies such as advertising on social media, emailing, printing flyers, offering incentives, and collaborating with relevant organizations are recommended when recruiting and retaining participants in evaluations of mHealth interventions [28-30]. In-person strategies should not be excluded where possible. For example, in-person strategies such as attending local events could provide participants with needed human interaction [28], acting as a guide to the mHealth intervention [29,30] and mitigating any concerns. However, advertising using social media platforms such as Facebook may be particularly useful in reaching individuals considered “hard to reach,” such as veterans [30-32]. Previous studies have shown that, while high rates of attrition still occur, recruitment for mHealth interventions using paid social media advertising may be more cost-effective and can target “hard-to-reach” populations effectively [32-34]. Thus, social media could help counter recruitment challenges faced during feasibility trials.

In addition, Pickering and Blaszczynski [35] have raised concerns regarding the quality of data and increased risk of bias from gambling research using online crowdsourcing platforms such as Amazon Mechanical Turk [36], Qualtrics [37], and Prolific [38]. However, Russell et al [39] argue that they can be effective in recruiting specific hard-to-reach groups given that stringent data quality checks are conducted to reduce potential biases [39]. These checks could include eligibility screening and validation of data quality such as military service numbers, which are identifiers unique to military populations. Prolific can provide high-quality data while being a cost-effective recruitment strategy [40,41]. Therefore, the combination of online crowdsourcing, social media advertising, and traditional recruitment (face-to-face meetings, emails, and flyers) may be effective for recruiting hard-to-reach and vulnerable populations such as military veterans in the United Kingdom with comorbid diagnoses of PTSD and harmful gambling (gambling disorder). However, to date, little is known about the relative effectiveness of these recruitment strategies.

In this study, we sought to compare these recruitment and retention methods for a pilot study designed to evaluate a 10-week app-based ACT intervention for UK military veterans experiencing PTSD and harmful gambling. Veterans were screened for PTSD severity using the PTSD Checklist for the *Diagnostic and Statistical Manual of Mental Disorders, Fifth Edition* (*DSM-5*) [42], with scores of 20 or above conferring study eligibility. Veterans experiencing harmful gambling were screened using the Problem Gambling Severity Index [43], with scores of 1 or above conferring eligibility, indicating some form of harmful gambling. Drawing on the work of Williamson et al [32] conducted on UK military veterans and alcohol use, the aim of this paper is to define and reflect on the recruitment strategies and make recommendations for future studies recruiting and retaining military veterans with co-occurring PTSD and harmful gambling.

## Methods

### Ethical Considerations

The recruitment strategies used in this study formed part of a larger study approved by the Swansea University School of Psychology Research Ethics Committee (1-2023-6798-6614). All participants provided written informed consent to take part. The data obtained were fully anonymized by the research team, and all participants were compensated with either Prolific funds or online shopping vouchers on completion of the larger study.

### ACT Vet

We developed the ACT Vet app using a co-design approach with veterans with lived experience of harmful gambling or PTSD and then adopted the Ionic Capacitor framework to achieve a research-viable product standard. ACT Vet is a self-guided app addressing the core components of ACT through seven modules: (1) an introduction that provides an overview of ACT Vet and ACT as a therapeutic practice, (2) Exploring thoughts around coping (cognitive defusion), (3) Control as the problem (acceptance), (4) Contact with the present (present moment awareness), (5) The self as the observer (self as context), (6) Living by your values (values), and (7) Committing to action (committed action ). The modules took approximately 15 minutes to complete, with a mandatory break period of 5 days taking place between completed and new modules. The ACT-specific module content took approximately 4 weeks to complete, and participants were encouraged to use the app for the remainder of the 10-week study.

### Online Recruitment

**Figure 1.**
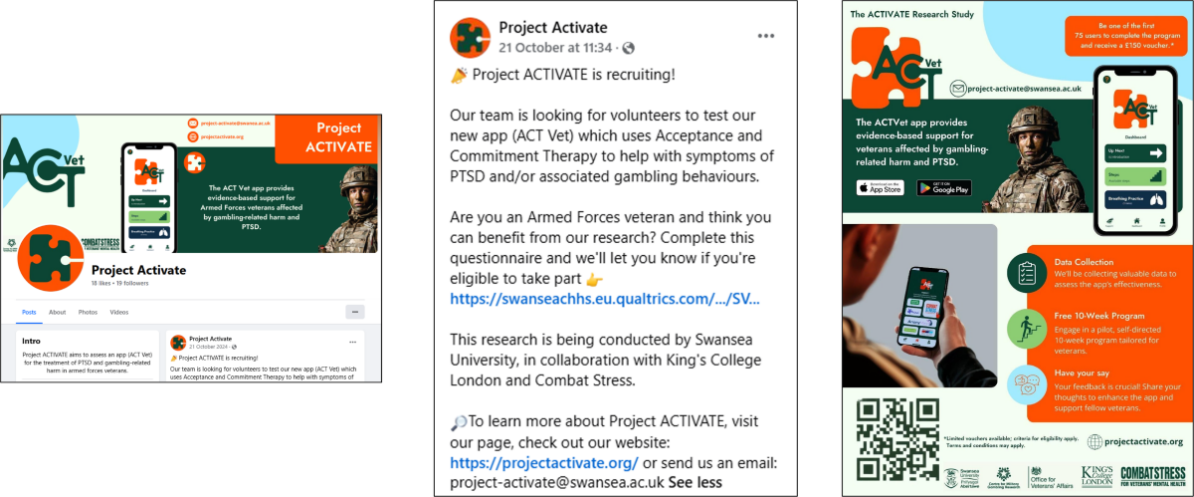
Screenshots from the ACT Vet app: (left panel) Facebook project page, (middle panel) paid advertisement, and (right panel) flyer for ACT Vet.

The primary recruitment and retention strategies included online engagement, providing study details and links across social media sites: Facebook, X (formerly known as Twitter), and Instagram. Individual project pages across each site (Facebook, X, and Instagram) were created (in May 2024) with the purpose of information sharing using blog-type posts and project recruitment (Figure 1, left panel). Paid Facebook advertisements were used as they have been considered a useful tool in similar veteran-specific app-based recruitment efforts [32]. These advertisements were run for a total of 27 days. The textual keywords that the target audience needed to have on their profiles to be included are as follows: Armed Forces Day (United Kingdom), veterans’ benefits (military) or army men, school or university, British Army and employers, Royal Air Force Regiment, Royal Marines, veteran, His Majesty’s Armed Forces, and Royal Navy or British Army. Figure 1, middle panel shows the imagery and copy used for the advertisement.

Additionally, Prolific, which is an online recruitment platform where researchers advertise studies among a large pool of vetted participants, was used to screen potential participants who were UK military veterans aged ≥18 years. A dedicated project-specific website was also launched that housed information and links related to participating in the study.

### Other Recruitment Strategies

Email addresses were collected from organization websites, and study details were sent. The research team carried out face-to-face meetings and visits with organizations such as the Forces in Mind Trust, National Health Service West Midlands Gambling Harms Clinic, the Royal British Legion, Combat Stress, Help for Heroes, Beacon Counselling Trust, GamFam, and GamCare. Physical and digital flyers (Figure 1, right panel) were created and circulated among organizations. Members of the research team also had personal contacts they used, including details of veterans who had previously agreed to participate in the study. Any veteran who agreed to take part was asked to encourage others who may have benefited from participating (snowball sampling).

The information regarding the recruitment source for each participant was documented and assigned a unique identifier, allowing the research team to track where participants had viewed and accessed the prescreening questionnaire (eg, Facebook, Prolific, or website). If eligible, previous military service numbers were also confirmed for the identification of spam or bots to ensure the integrity of the data. Unless provided by the recruitment platform, demographic information was collected at the baseline time point. Finally, it was advertised that, on completion of the 10-week program, each participant would be offered a high street voucher of £150 (US $189.77) for their time.

### Measures and Analysis

Our primary outcome measures included advertisement duration in days, cost in GBP (and the USD equivalent), online market reach or armed forces audience criteria, online post link clicks, and online post shares and reactions. Please note that online market reach differs depending on when digital marketing campaigns are conducted because of the underlying Facebook or Meta analytics. Means and SDs were reported for the continuous variable (respondents’ ages), and percentages and frequencies were reported for categorical variables.

## Results

### Online Recruitment

#### Facebook

From the dedicated Facebook page, posts were circulated in veteran and gambling-related groups. Paid advertisements were also circulated separately for a total of 27 days to reach veterans or those with connections to veterans aged 18 to 65 years in the United Kingdom. Facebook campaign key performance indicators provide the performance and cost breakdown of each advertisement campaign and are shown in Table 1.

**Table 1.** Key performance indicators (KPIs) for ACT Vet paid Facebook advertising.

KPI	Paid advertisement campaign number		
	1	2	3
Advertisement duration (days)	3	14	10
Cost, £ (US $)	5.00 (6.33)	100.00 (126.51)	177.70 (224.81)
Total reach (number of people)	1132	14,682	117,085
Number of link clicks	61	506	854
Cost per click, £ (US $)	0.08 (0.10)	0.20 (0.25)	0.21 (0.27)
Number of post shares	2	14	1
Number of post reactions	1	12	2

The first paid campaign was run as a test for 3 days and reached 1132 people (n=112, 9.89% female; n=1020, 90.1% male), of whom 340 (30.03%) were aged between 35 and 44 years, with 1114 (98.41%) viewing it on mobile devices. The second advertisement campaign reached a total of 14,682 people (n=3656, 24.9% female; n=11,026, 75.1% male), of whom 4405 (30%) were aged between 55 and 64 years, with 12,263 (83.52%) viewing it on mobile devices. The third advertisement campaign reached a total of 117,085 people (n=70,836, 60.5% female; n=46,249, 39.5% male); 35,126 (30%) were aged ≥65 years, with 67,326 (57.5%) being reached using the audience network (third-party apps such as Instagram or mobile games).

Facebook accounted for 7 participants who were eligible and consented through organic (without paid advertising) page or post interactions, with an additional 14 who were eligible and consented through paid advertising, which cost £20.19 (US $25.55) per consenting individual. Recruitment outcomes from paid Facebook advertising are shown in Table 2. In total, Facebook accounted for 21 eligible participants. Unless provided by the recruitment platform, demographic information was only collected at the baseline time point.

**Table 2.** Total recruitment outcomes across paid Facebook advertising and Prolific.

	Paid Facebook advertising	Prolific
Total cost, £ (US $)	282.70 (357.65)	157.50 (199.25)
Number of clicks or submissions	1421	71
Cost per click or submission, £ (US $)	0.49 (0.62)	0.75 (0.95)
Number of eligible consenting individuals	14	50

#### X (Formerly Known as Twitter)

Additionally, a dedicated X page was created and amassed 170 followers. The page consisted of 32 posts related to recruitment advertising and blog-type posts showing the research team’s activities. The highest engagement was with a blog-type post with 419 views, followed by a recruitment advertisement with 328 views. No participants were directly recruited.

#### Instagram

An Instagram page was also created, which resulted in 16 followers. The page consisted of 4 posts designed to raise awareness of the project and recruit participants. The highest engagement was through 6 likes on a post circulating project awareness. No participants were directly recruited.

#### Prolific

The study also used Prolific. The built-in screening criteria of “UK military veteran” and “aged ≥18 years” were applied, yielding a total of 287 eligible participants who had been active in the previous 90 days on Prolific. The study recruitment target was set at 150 participants. Sensitive content warnings were given as the study involved topics related to mental health. Participants were paid £0.75 (US $0.95), the equivalent to £9.00 (US $11.39) per hour, for the 5 minutes it took to complete the prescreening questionnaire. The total cost (including fees and value-added tax) for the screening was £157.50 (US $199.25). From this screening, 71 submissions were made (n=63, 89% male; n=8, 11% female), with a mean age of 45 (SD 13.24) years. Of these 71 participants, 50 (70%) met the eligibility criteria and consented to be sent app codes, which cost, on average, £3.15 (US $3.99) per consented individual. Table 2 shows the total recruitment outcomes from Prolific. Recruitment ceased without meeting the target of 150 participants due to time constraints.

#### Website

The project-specific website (including domain hosting) was used as a platform for project dissemination and recruitment and was launched on January 8, 2024, and continued to function until the project end. Four eligible participants completed the prescreening.

#### Email

After collecting email addresses from organization websites, nearly 200 emails were sent. One eligible participant was recruited and consented to be sent app codes.

#### Face to Face

Throughout recruitment, a total of 15 veteran, charity, or clinical meetings (in person and online) related to military veterans or gambling across the United Kingdom were conducted. Contact with organizations was vital to raise awareness of the study and interact with the veteran community. One eligible participant consented to be sent app codes directly from a face-to-face meeting, whereas another participant consented to be sent app codes via word of mouth after a meeting.

#### Unknown

One participant accessed the prescreening questionnaire directly, and the recruitment strategy could not be identified.

### Study Attrition and Sample Characteristics

**Figure 2.**
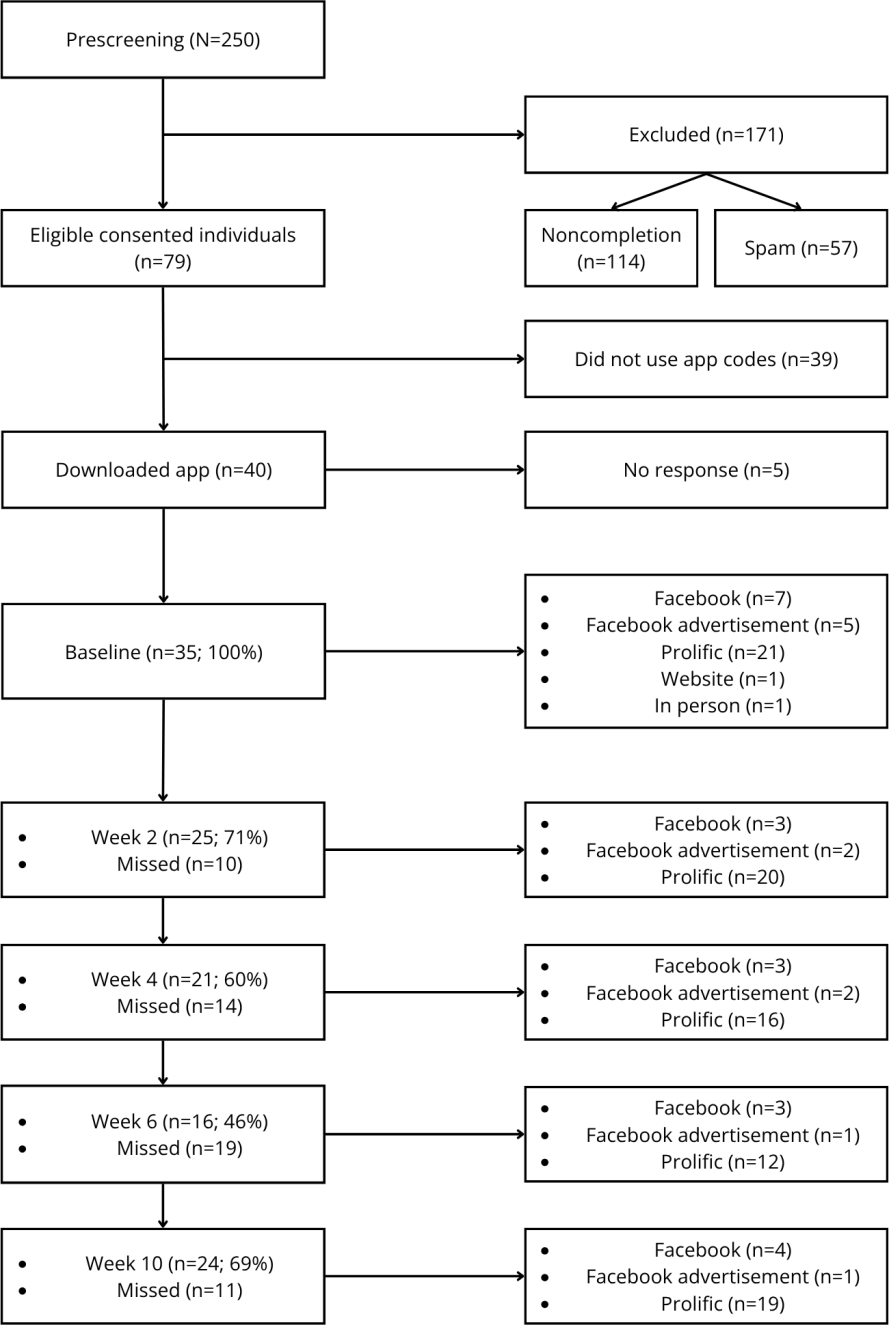
Study attrition and recruitment sources across project time points. “Facebook advertisement” indicates paid advertising compared to unpaid Facebook recruitment.

Figure 2 shows the attrition from initial screening to week 10, and the completed time points are split by recruitment source. A total of 35 participants (n=31, 89% male; n=3, 9% female; and n=1, 3% undisclosed) completed in-app baseline time points, and 1 (3%) participant formally withdrew from the study. The mean age was 44.77 (SD 10.89) years, and all participants (35/35, 100%) were of White British ethnicity, with 49% (n=38) having a diagnosis of PTSD and none having a *DSM-5* diagnosis of gambling disorder. A total of 24 participants (n=21, 88% male; n=2, 8% female; and n=1, 4% undisclosed) with a mean age of 45.29 (SD 10.70) years finished the week 10 time points and had a mean PTSD Checklist for the DSM-5 score of 40.92 (SD 20.12) and mean Problem Gambling Severity Index score of 5.47 (SD 3.59), indicating moderate risk of problem gambling. According to responses to the mHealth App Usability Questionnaire, participants provided high ratings of usability (mean 6.09, SD 0.76; range 4.18-7.00), indicating that the app was liked by veterans.

## Discussion

### Principal Findings

This paper outlines recruitment and retention strategies used for ACT Vet, a smartphone digital intervention for UK military veterans with PTSD and harmful gambling. Overall, Facebook (both unpaid and paid advertising) and Prolific were the most effective strategies for recruiting eligible participants. The combined alternative recruitment methods (hub visits, meetings, emails, and website) allowed for building interdisciplinary networks among researchers, service users, and practitioners but resulted in few participants. Challenges throughout recruitment remained and included low conversion rates from social media advertising, spam or bots, ineligible participants, participant attrition, and missing questionnaire time points. However, app usability ratings were high, and the app was well received.

Social media was effective in recruiting eligible participants, aligning with previous findings [30,32-34]. In this study, although we only ran brief advertisement campaigns, Facebook outperformed traditional strategies in the recruitment of eligible veterans. However, there was a noteworthy difference in link clicks through to eligible consent using paid Facebook advertising. Most of those who showed initial interest by clicking the advertisement link (n=1421) did not proceed through to eligible consent (n=1407, 99.01%). Even so, as evidenced by these results and highlighted by Aily et al [33], a low conversion rate from Facebook paid advertising can still contribute to a cost-effective recruitment strategy.

Prolific was also effective in recruiting and retaining eligible military veterans. Most of those who completed final questionnaire time points were recruited via Prolific (n=19, 79%) and helped the study access secondary categories of groups that may be hard to reach [39]. Prolific was also the most cost-effective compared to all other strategies. This is in line with previous work that argues that Prolific can be cost-effective and reliable for high-quality data [40,41]. Hence, our study shows that the combination of Facebook and Prolific is important in recruiting for clinical research, particularly military veterans with co-occurring PTSD and harmful gambling.

### Challenges

Notwithstanding the aforementioned advantages to the approaches used, we did encounter some recruitment and retention challenges. Few veterans were recruited through strategies such as face-to-face meetings, emails, or visits to organizations. Veterans can take up to 11 years to access support services and may face significant barriers and stigmatization when accessing treatment [1,3], which may have contributed to the limited interaction observed in this study. However, due to the anonymous nature of this study, it was difficult to measure whether more veterans who later accessed the online information were recruited because of our in-person visits. For this reason, it is advisable that future digital or online interventions maintain a physical presence among service users, charities, and hubs to build in-person networks and raise further awareness. Moreover, future recruitment initiatives should consider ensuring that long-term strategies and resources are dedicated to developing engaging content and other in-person activities with veterans.

In addition, the reliance on social media resulted in many spam responses and unreliable data, which may have contributed to the large attrition rate. Even among eligible participants, close to half (n=39, 49%) did not respond after requesting and being sent individualized app codes and did not go on to complete baseline measurements. These participants could not be contacted and, therefore, were considered to have withdrawn. Similar instances of attrition have been noted in UK-based military veteran digital interventions that have used social media for recruitment [19,20] and are considered a key challenge in clinical mHealth research. As recommended by Parkes et al [20], future work using social media for recruitment should also ensure that additional strategies and resources are made available to counteract any attrition from social media recruitment.

Data attrition inevitably resulted when participants did not complete required questionnaires when requested. This led to variation between program time and data points. In this study, there was an overall decrease in completion of all measures from baseline to each time point, yet the final week, week 10, had more completions than interim weeks such as weeks 4 and 6. One reason for this could be that participants stopped using ACT Vet after all steps had been completed within the app by the week 4 time point. While push notifications and email reminders were sent, this study still experienced a decrease in completed measures during those weeks. The opportunity to provide week 10 data was provided using an email link after a lengthy period despite also being the final time point, which may have resulted in an increased response rate. Thus, if the program length extends past the intervention length (ie, in study designs with extended follow-up), attrition is likely to occur without targeted initiatives to increase engagement.

A final retention challenge may have inadvertently stemmed from offering remuneration in a single large amount at the end of the study. That is, attrition may have been reduced had we adopted a progressive or escalating schedule of payments similar to those used in research on incentivized treatment attendance or adherence to interventions for related addictive behaviors [44]. This possibility warrants consideration in future mHealth intervention research.

### Limitations

There were some limitations to the recruitment and retention methods we used. First, participants recruited through Prolific were self-selected, which may increase the likelihood of bias and decrease the likelihood of obtaining a representative sample [35]. Despite this, data integrity measures were implemented throughout, such as requiring service numbers as evidence of veteran status and validating data quality. Second, as found in similar recruitment efforts, the use of online media platforms may have excluded veterans without social media or with limited experience using technology [32]. However, this study implemented mitigating strategies to reduce these issues [30]. Third, the range of colors, imagery, and designs used for the project-specific branding may have resembled army branding (ie, army green and camouflage patterns). There is a possibility that this branding impacted recruitment of veterans from other services such as the Royal Navy and the Royal Air Force, both of which have unique colors and branding. Finally, 89% (n=31) of all eligible recruited participants were male, which resulted in an underrepresentation of female veterans. Although female representation in the regular UK armed forces increased by 11.7% in 2024 [45], the veteran criteria for ACT Vet likely resulted in a sample that reflected representation in the UK armed forces, as previously noted by Williamson et al [32]. Furthermore, men are considered at high risk of harmful gambling [46], which may have contributed to the sex variance observed.

### Conclusions

In conclusion, our findings showed the effectiveness of social media and online platforms compared to additional recruitment strategies in line with previous findings and recommendations for mHealth recruitment [30,33,34], particularly for military veterans [32]. Furthermore, these findings can be extended to populations with comorbid conditions, such as veterans with both PTSD and harmful gambling. In this paper, we offer some key recommendations for future recruitment efforts. First, face-to-face meetings and visits should be organized as early as possible to strengthen interdisciplinary relationships and networks, but they should not be solely relied upon for recruitment. Second, a strong online presence should be built across various platforms, and unpaid and paid strategies should be used. This will assist in project awareness and recruitment from populations that may be hard to reach, such as those with PTSD and harmful gambling. Finally, increased efforts should be made to diversify branding and advertising. For military veteran research, this should reflect all 3 UK regular armed forces services (Army, Royal Navy, and Royal Air Force), which, in turn, may also lead to an increase in participation from female veterans with comorbid disorders. It is hoped that these findings inform future recruitment initiatives in veteran and gambling research and mHealth research more broadly.

## Abbreviations

ACT: acceptance and commitment therapy
*DSM-5*: *Diagnostic and Statistical Manual of Mental Disorders, Fifth Edition*
mHealth: mobile health
PTSD: posttraumatic stress disorder
